# Cardiovascular medtech: the grand challenge of computer simulations

**DOI:** 10.3389/fmedt.2023.1304223

**Published:** 2023-11-13

**Authors:** Stéphane Avril

**Affiliations:** Mines Saint-Etienne, Université Jean Monnet, INSERM, U 1059 Sainbiose, Saint-Etienne, France

**Keywords:** digital twin, endovascular intervention, mini-invasive, simulation—computers, aneurysm

With the major progress of imaging techniques such as 3D angiography, and the development of navigation systems in recent decades, minimally invasive cardiovascular procedures have continuously taken ground from open surgery, inducing benefits for patients like faster recovery, reduced pain, and lower risk of complications. For instance, in the management of cerebral aneurysms, endovascular treatment has become the preferred choice, whereas open surgery remains an option for the treatment of ruptured aneurysms with compressive haematomas or aneurysms that cannot be accessed via endovascular route. The trend is similar in the cardiac and aortic territories, where the market size of transcatheter implantable devices, such as aortic valves, endografts for thoracic and abdominal aneurysms, peripheral stents and coronary stents, have been continuously increasing. Nowadays, just as an example, about 75% of aneurysm aortic repair in Europe and in the United States are endovascular (1).

The boom of mini-invasive procedures has been followed by the need to predict more and more precisely the behavior of increasingly complex cardiovascular devices inserted and deployed in each patient's arteries, veins, or heart. Mini-invasive interventions require the acquisition of 3D scans, usually obtained via x-ray computed tomography (CT), in order to plan the intervention, assess the device dimensions for each patient, support device selection and sizing, and define the scenario of intervention. With these pre-operative imaging and planning operations integrated into the interventional workflow, the “cardiovascular medtech” sector has fully entered the world of personalized and precision medicine, which involves managing lesions based on each individual characteristic. This represents a big challenge in terms of required resources given the increasing number of patients with the aging population. Moreover, if we take for instance the example of aortic aneurysm repair, endovascular procedures have become technically time- and contrast-consuming and require significant experience and advanced skills, with a substantial learning curve.

Simultaneously with the development of devices for mini-invasive interventions, another increasingly popular technology in “cardiovascular medtech” is the technology of digital twins. Initially proposed in 2003 by NASA, it was defined as a computer simulation of a system that uses the best available physical models, sensor updates, data from other systems, etc., to represent the behaviour of the actual system. In medicine, digital twins have already led to industrial applications in the following domains:

A. Digital twins for virtual and/or remote medicine. They consist of computational models of physical phenomena (e.g., blood fluid dynamics, device and tissue deformations, electromechanics for the heart) in personalized anatomies (2). Working prototypes have already been deployed by start-up companies in cardiovascular applications, i.e. the start-up Sim&Cure has established a digital twin platform for brain aneurysms, the start-up Feops a digital twin platform for transcatheter aortic valve replacement and the other start-up Predisurge a patient-based digital twin platform for endovascular aortic aneurysm repair.
B. Digital twins for data fusion for patient monitoring and diagnostics. Major academic initiatives have been undertaken to gradually include novel tailored computer-enabled decision points (3). Siemens Healthineers, for instance, has also developed a software that generates a digital twin of a patient's heart. Remote diagnostics based on AI technology are now used to accurately and instantly predict heart disease risk through non-invasive, ultrasound analysis, for instance, to diagnose heart disease coronary.
C. Digital twins for health management, prevention, and well-being: Ross has presented work relying on AI algorithms with Hewlett-Packard to create digital twins in different ways (4). A particular very lively technology is related to smart homes systems. However, there is generally no mechanistic model that can relate the types of data monitored over the day-to-day life and the impact of our lifestyle.

Digital twins of type A, i.e., personalized computational models, have a very high potential for mini-invasive “cardiovascular medtech”, as they can provide aid in performing pre-operative steps and assist interventionists during the intervention, reducing the increasing weight on resources and providing security similar to highly-skilled interventionists (2, 5). However, the use of digital twins in clinical routine for mini-invasive cardiovascular procedures is still in its infancy. A situation that we can anticipate in the next 10 years is that simulating computationally a mini-invasive cardiovascular intervention would become a service offered to every patient and would be mandatory for securing the most complex interventions (10%–20% due to tortuous anatomies or calcifications for instance) or for the clinical studies related to new medical devices (in silico trials).

To reach this stage, a considerable research effort is still necessary to make computer simulations more reliable and accessible to interventionists in any type of cardiovascular procedure. The following research directions should be considered:

1. Coupling computer simulations with Internet of Things (IOT). For example, recent innovations such as smart balloon catheter systems, which are equipped with electronics, sensors, and mechanisms, are constantly being redesigned to deliver ablation therapy, blood flow data, hematologic data, and electrical stimulation. These sensors can feed computational models with data to improve their predictions. Moreover, computer simulations could be used to update the position of endovascular devices equipped with 3D magnetic position sensors.
2. There is an urgent need for thorough and comprehensive *verification, validation, and uncertainty quantification* of computational models. Regulators now consider also evidence produced in silico, but accepted methods are needed to evaluate the credibility of models, such as the use of the ASME V&V-40 technical standard. A major source of uncertainty is the properties of tissues, requiring new imaging systems that can assess them non-invasively (6).
3. The potential of computer simulations is also for more tailored devices in “cardiovascular medtech”. The development of virtual surrogates for hemodynamic and structural analyses, such as the Living Heart Project of Dassault Systèmes, enables experiments and testing of new devices in virtual patients.
4. Computational models currently predict the intra-operative effects and the post-operative outcome, but there is a need for the prediction of long-term complications. For instance, the effects of blood flow on the deployed stents or coils should be investigated by fluid-structure interaction (FSI) simulations. In particular, this is essential for anticipating device migrations, endoleaks, thrombosis, and other complications.
5. Numerical models in cardiovascular biomechanics can be very expensive computationally and reduced-order models have to be developed to decrease this computational cost, with the objective of reaching real-time simulations. However, the geometric complexity and the anatomical variability combined with the non-linearity issues lead to the “curse of dimensionality”, and thus pose a challenge to standard data-driven models.
6. Machine learning (ML) in Artificial intelligence (AI) will soon enable automatizing the preparation of personalized computer models. Moreover, it is technically possible to achieve real-time simulations with ML models trained with standard finite-element simulations (7). Therefore, AI is expected to be used in all the computer simulations for “cardiovascular medtech”. However, a number of challenges are still unsolved: health data can be of variable quality, or incomplete, making it difficult to collect large databases for training ML models. In addition, the use of ML in clinical practice raises complex issues related to transparency and privacy. Addressing these challenges still requires continuous research efforts in collaboration with healthcare professionals, regulators and AI developers.

In summary, this Speciality Grand Challenge is a personal viewpoint on some of the key questions in the broad cardiovascular medical technology domain, with a focus on innovations and research related to computational modelling. More details on the applications can be found in the cited references. Other important challenges are underlined in the mission and scope of the section, which aims to promote and disseminate the very best quality research in “cardiovascular medtech”.
